# Mutation analysis of the *MSMB* gene in familial prostate cancer

**DOI:** 10.1038/sj.bjc.6605485

**Published:** 2009-12-08

**Authors:** Z Kote-Jarai, D Leongamornlert, M Tymrakiewicz, H Field, M Guy, A A Al Olama, J Morrison, L O'Brien, R Wilkinson, A Hall, E Sawyer, K Muir, F Hamdy, J Donovan, D Neal, D Easton, R Eeles

**Affiliations:** 1 Translational Cancer Genetics Team, The Institute of Cancer Research, 15 Cotswold Road, Sutton, Surrey SM2 5NG, UK; 2 Department of Oncology. University of Cambridge, Strangeways Laboratory, Worts Causeway, Cambridge CB1 8RN, UK; 3 CR-UK Genetic Epidemiology Unit, University of Cambridge, Strangeways Laboratory, Worts Causeway, Cambridge CB1 8RN, UK; 4 University of Nottingham Medical School, Queens Medical Centre, Nottingham NG7 2UH, UK; 5 Nuffield Department of Surgery, University of Oxford, Oxford OX3 9DU, UK; 6 Department of Social Medicine, University of Bristol, Canynge Hall, Whiteladies Road, Bristol, UK; 7 Surgical Oncology (Uro-Oncology: S4), Departments of Oncology and Surgery, University of Cambridge, Box 279, Addenbrooke's Hospital, Hills Road, Cambridge CB2 2QQ, UK; 8 Cancer Research UK Cambridge Research Institute & Li Ka Shing Centre, Robinson Way, Cambridge CB2 0RE, UK; 9 The Royal Marsden NHS Foundation Trust, Downs Road, Sutton, Surrey SM2 5PT, UK & Fulham Road, London SW3 6JJ, UK

**Keywords:** *MSMB*, prostate cancer, SNP, *in silico*, gene expression

## Abstract

**Background:**

*MSMB,* a gene coding for *β*-microseminoprotein, has been identified as a candidate susceptibility gene for prostate cancer (PrCa) in two genome-wide association studies (GWAS). SNP rs10993994 is 2 bp upstream of the transcription initiation site of *MSMB* and was identified as an associated PrCa risk variant. The MSMB protein is underexpressed in PrCa and it was previously proposed to be an independent marker for the recurrence of cancer after radical prostatectomy.

**Methods:**

In this study, the coding region of this gene and 1500 bp upstream of the 5′UTR has been sequenced in germline DNA in 192 PrCa patients with family history. To evaluate the possible effects of these variants we used *in silico* analysis.

**Results:**

No deleterious mutations were identified, however, nine new sequence variants were found, most of these in the promoter and 5′UTR region. *In silico* analysis suggests that four of these SNPs are likely to have some effect on gene expression either by affecting ubiquitous or prostate-specific transcription factor (TF)-binding sites or modifying splicing efficiency.

**Interpretation:**

We conclude that *MSMB* is unlikely to be a familial PrCa gene and propose that the high-risk alleles of the SNPs in the 5′UTR effect PrCa risk by modifying *MSMB* gene expression in response to hormones in a tissue-specific manner.

Prostate cancer (PrCa) is the most common cancer in men in the western world, with 34 000 new cases every year and a lifetime risk of 1 in 14 in the United Kingdom (Cancer Research UK Factsheets, 2008). However, its aetiology remains poorly understood. The substantial worldwide variation in incidence rates suggests that there are lifestyle risk factors, but none have been identified definitively. Apart from demographic factors, the only well-established risk factor for PrCa is family history. The risk of the disease in first-degree relatives of cases is approximately twice that of the general population (Carter *et al*, 1992; Goldgar *et al*, 1994; Eeles, 1999; Hemminki and Czene, 2002; Gronberg, 2003; Edwards and Eeles, 2004). Familial risk is four-fold greater amongst close relatives of cases under 60-years old. Men with two or more affected relatives are at even higher risk. Analyses of the Nordic twin registries show higher risks in monozygotic compared with dizygotic twins, thereby supporting the hypothesis that much familial aggregation is due to genetic factors rather than shared lifestyle factors (Lichtenstein *et al*, 2000). Epidemiological studies consistently demonstrate aggregation of PrCa in families, consistent with a multi-genetic origin.

To identify some of the multiple susceptibility loci we recently carried out a genome-wide association study (GWAS) of ∼550 000 single base pair genetic variants (SNPs) in 1854 PrCa cases and 1894 controls. Seven new susceptibility loci were validated in a further set of 3650 PrCa cases and 3940 controls containing several plausible candidate genes, one of which was on chromosome 10 (Eeles *et al*, 2008). Single base pair genetic variants rs10993994 and rs7920517 lie within an LD block of ∼100 kb on chromosome 10, containing the *β*-microseminoprotein beta gene, *MSMB*. The most strongly associated SNP, rs10993994, lies 2 bp upstream of the transcription start site of *MSMB*. This association was also reported by the CGEM study (Thomas *et al*, 2008). *MSMB* codes for PSP94, a prostatic secretory protein, synthesised almost exclusively in the prostate gland and it is the major constituent of seminal plasma. PSP94 functions in growth regulation and induction of apoptosis in PrCa cells (Garde *et al*, 1999) and, as it leaks into the blood, its serum level can be measured. There is a correlation between a reduced level of PSP94 and PrCa progression (Reeves *et al*, 2006; Bjartell, 2007), after radical prostatectomy. Thus, it is clear that the regulation of the expression of *MSMB* is a key element in PrCa development and any sequence variant, which has an effect on the level of *MSMB* gene expression would be a good candidate for a causal variant.

The location of the rs10993994 and the strength of the association (*P*=10^−17^) raise the possibility that this SNP may be causally related to disease risk, although this remains to be proven. However, GWAS are designed to tag common variants, and associations mediated by rare variants may have been missed. In order to establish the contribution of variants at this locus to familial PrCa and to explore the possibility that there may be additional disease-associated variants in the *MSMB* gene, we re-sequenced the genomic sequence of the *MSMB* gene including a ∼1500 bp region upstream of the transcription start site in 192 PrCa cases with strong family history of the disease.

## Materials and methods

Whole blood samples from PrCa cases were collected as part of the UK Genetic Prostate Cancer Study (UKGPCS) at the Institute of Cancer Research (http://www.icr.ac.uk). We have selected 192 families with three or more cases of PrCa. A sample from one person per family was used for sequence analysis and wherever possible this was the youngest family member affected with PrCa. Control samples were from the ProtecT study; this is a national study of community-based PSA testing and a randomised trial of subsequent PrCa treatment (Donovan *et al*, 2003). Men between the ages of 50 and 69 years are being recruited through general practices in nine regions in the UK. DNA was extracted from their peripheral blood using standard methods as described previously (Eeles *et al*, 2008).

For the familial cases the full coding sequence of the *MSMB* gene, exon–intron boundaries and a ∼1500 bp region of the 5′UTR region was analysed by sequencing using the BigDye Terminator Cycle Sequencing kit (v3.1) and a 3730*xl* DNA Analyzer, (ABI Perkin Elmer, Foster City, CA, USA). Control samples were sequenced only for the 5′UTR region to assess the allele distribution of the newly discovered promoter SNPs. One new variant, rs12770171 was analysed by the 5′nuclease assay (Taqman) using the ABIPrism 7900HT sequence detection system according to the manufacturer's instructions. Primers and probes were supplied directly by Applied Biosystems, Foster City, CA, USA (http://www.appliedbiosystems.com/) as Assays-By-Design.

To identify the potential effects of sequence variants in the promoter and intronic regions, 161 nucleotide sequences around each SNP were taken from Ensembl (FASTA) and the alternative alleles inserted. These sequences were submitted to GenomatixSuite MatInspector, which offers the most complete library available for transcription factor (TF)-binding sites (Cartharius *et al*, 2005) and we also applied a tissue filter specific for prostate. Associations between SNP genotypes and PrCa risk were tested using a Cochrane–Armitage trend test and genotype-specific risks were estimated as odds ratios (ORs) with associated 95% confidence interval (95% CI). For Hardy–Weinberg equilibrium and Armitage trend testing, we used the public software developed by Tim M Strom and Thomas F Wienker (http://ihg.gsf.de/cgi-bin/hw/hwa1.pl). For the haplotype analysis we used Haploview (Barrett *et al*, 2005) and Haplo.Stats (Schaid *et al*, 2002).

## Results

We have sequenced the *MSMB* gene and a 1500 bp 5′UTR region in 192 blood DNA samples with strong family history (⩾3PrCa cases in the family). No deleterious mutation was found in any of the exons, but we identified nine new SNP sequence variants as well as six other previously known SNPs in HapMap. The list of all the SNPs in this region is shown in Table 1.

Four of the new variants are in the 5′ UTR of the *MSMB* gene, these were found in addition to six previously known SNPs in this region. This region has been characterised previously as the proximal promoter region for *MSMB*. In all, 10 out of 17 SNPs identified lie in the promoter region. Of this region, 1500 bp was resequenced in 192 control samples to analyse the relative frequency of the three commonly known SNPs in the 192 PrCa cases and 192 control samples (Table 2a). SNP2 (ENSSNP10237085), SNP8 (rs12770171) and SNP9, (rs1093994) all were significantly associated with PrCa risk. Single base pair genetic variants was a previously uncharacterised SNP (it was not genotyped in HapMap Phase 2). To further investigate its association with PrCa risk, we genotyped blood DNA from 3268 cases and 3366 controls. We found strong evidence for an association between rs12770171 and PrCa risk (*P*=1.41 × 10^−12^), however, this SNP is in LD with rs10993994 (*r*^2^=0.32) and multiple logistic regression and haplotype analysis revealed that there was no evidence for an independent association with rs12770171 after adjustment for rs10993994 (Table 3).

**Figure 1:**
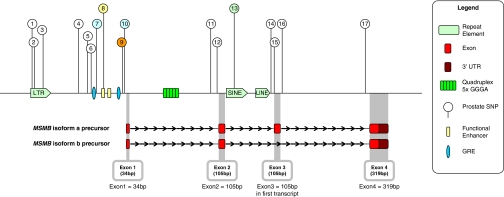
Physical disposition along chromosome 10 of new SNPs from prostate cancer (PrCa) patients, showing previously characterised glucocorticoid-responsive elements and enhancers, transcripts and repetitive elements. *In silico* analysis showed that SNP7 is predicted to alter the response to glucocorticoid transcription factors (TFs) in prostate tissue; SNP8 is the most conserved and falls within an enhancer; SNP9 (rs10993994) is predicted to change the binding site for the ubiquitous CCAAT and Gli–Kreupel TFs, whereas only the common allele of SNP10 is predicted to bind homeobox TFs. SNPs 13 and 14 are also highly conserved; binding of splice factors is predicted to be altered by SNP14 alleles.

Functional relevance of the sequence variants was assessed *in sili*co, by examining the regions around the 17 SNPs for conservation and allele-specific splice factor or TF binding. SNP8 lies in a biochemically characterised enhancer region of the promoter (−275 to −206 upstream of the start ATG, Ochiai *et al*, 1995) but no TF is predicted to bind across SNP8. SNPs 7, 8, 9 (the original best hit) and 10 are all in a 400 bp region, ending in the ATG transcription start site. Of the four SNPs, 8 and 9 lie in the best-conserved sequence, with SNP8 being the best conserved SNP across mammals (Supplementary Table 1). SNP9 (rs10993994) is predicted to change the binding site for the ubiquitous CCAAT and Gli–Kreupel TFs. SNPs 7 and 10 are predicted to have allele-specific TF binding in prostate tissue. SNP7 is predicted to bind glucocorticoid receptor TFs, including androgen and progesterone receptors, NR3C1&2 (nuclear receptor subfamily 3, group C) and aldosterone-receptor TFs. The rare allele of SNP7 (c.-238 C>T) increases predicted glucocorticoid binding two-fold, and is predicted to displace binding of ubiquitous E-box TFs (including Myc). The common allele of SNP10 (c.-19 T>G), is predicted to bind NKX homeobox domain TFs. The *in-silico* data for SNPs7–10 are summarised in Figure 1.

Glucocorticoid TF-binding sites are also found across SNP15 and close to (within 50 bp of) SNP11/12, SNP14 and SNP16. Allele-specific alterations in binding of splice factors SFp40, ASP/SF2 are predicted for SNP12.

The two SNPs predicted to have prostate-tissue and allele-specific effects on TF binding are rare sequence variants; SNP7 has not been previously reported and we found it in only 1 out of 192 case samples (this variant was also present in a sibling with PrCa); SNP10, rs41274660, is found at a frequency of 7 out of 192 heterozygotes and 1 out of 192 homozygotes in our familial cases compared with 6 heterozygotes in 192 controls; therefore there is no evidence that this SNP is associated with PrCa risk.

## Discussion

We present the resequencing results of the *MSMB* gene and its 5′UTR region in familial PrCa cases and controls. Recently, two GWAS identified *MSMB* as a PrCa susceptibility locus. Both studies found that SNP rs10993994 is associated with PrCa risk, with a per allele OR of 1.25, *P*=10^−13 to −29^.

Resequencing germline DNA from 192 familial PrCa cases did not find any deleterious mutations in the coding region of *MSMB*, hence it is unlikely that this gene is altered by rare deleterious coding mutations in familial PrCa. We have identified nine new sequence variants and using bioinformatics tools, have assessed their predicted effect on *MSMB* gene expression/regulation. The *MSMB* gene consists of four exons and is located on chromosome 10q11.2. In the upstream region of *MSMB* there are many putative transcription regulatory elements and it has been shown that the proximal promoter regions, −275–207 and −186–128, function in a prostate-specific manner. We have identified several new sequence variants in the non-coding intronic and promoter regions. SNP8, rs12770171, a previously uncharacterised SNP was found to be strongly associated with PrCa in our familial set, however, this association could be explained by the correlation between this SNP and rs10994993 and therefore it is not independently associated. *In silico* analysis revealed that SNP8 (rs12770171) lies within a known enhancer region and we propose that it might have an effect on gene regulation. The most strongly associated SNP, SNP9 (rs10993994) is predicted to change the binding site for the ubiquitous CCAAT and Gli–Kreupel TFs. *In vitro* studies by Buckland *et al* (2005) showed that both SNPs 8 and 9 have a substantial effect on the function of MSMB, reducing its activity by 60–70%. Some of the other SNPs are, however, also predicted to affect the expression level of *MSMB*, in particular, the very rare, high-risk allele of SNP7 amplifies the binding site for androgen and progesterone receptor in prostate tissue at the expense of a ubiquitous cell cycle/growth TF. As a result, a subtle alteration in the control of *MSMB* expression, changing ubiquitous cell-cycle/growth regulation to hormone regulation is predicted in the presence of the high-risk allele. As we found this alteration in only 1 out of 192 PrCa families, this variant could be a rare mutation predisposing to PrCa. SNP10 is predicted to bind NKX homeobox domain TFs and this would lead to allele-specific tissue specificity as well as hormonal regulation. Altogether as SNPs 7–10 are all predicted to influence the regulation of gene expression, their additive effects could result in a large variation in MSMB expression.

Yeager *et al* (2009), have reported the resequence analysis of a 97 kb region containing the *MSMB* gene using DNA from 70 individuals (36 PrCa cases and 28 controls). They identified 348 SNPs, of which 157 were new. Similarly for us, they did not discover any SNPs in perfect LD with rs10993994 or any coding SNPs in *MSMB*. As they only list the common SNPs (MAF>5%), which are catalogued in dbSNP we are unable to make a comparison with the less common SNPs or rare variants reported here. Considering the difference between the sample sets, it is likely that the rare variants found by us were not identified in the study of Yeager *et al* (2009).

In a recent functional study it was shown that the risk allele of SNP9, rs10993994, had only 13% of the promoter activity of the wild-type allele in the PrCa model LNCaP cell line and there was a dose–dependant increase in *MSMB* promoter activity in the wild-type allele with the synthetic androgen R1881 (Chang *et al*, 2009). In addition, Lou *et al* (2009) have also shown a significant effect of the risk allele on gene expression *in vitro* and that the non-risk allele preferentially binds to the CREB TF. This provides further evidence that rs10993994 (SNP9) is likely to be the strongest causative variant. However, the functional effects of all the above described variants on gene expression will need to be tested *in vivo* on samples whose DNA has been genotyped for all these SNPs. It is possible that allele-specific alterations in splicing are combined with effects on transcriptional levels. A SNP lying within a known, biologically active element is most likely to have an effect, so the *in silico* predictions for SNP9 agree with the laboratory observations.

Although a SNP within a biologically validated TF-binding site is a good indicator for the possible functional effect on transcription, it is known that a large proportion of functional SNPs do not lie within the known consensus of TF-binding sites. Functional SNPs may exert their effect by other mechanisms, such as changing the structure of the DNA or by affecting splicing. Ultimately one would need direct evidence of functionality from *in vitro*/*in vivo* models, but the collection of expression and segregation analysis data would also be helpful in this assessment.

The protein product of *MSMB* is PSP94, a small cysteine-rich protein. It is abundantly expressed in seminal fluid, possibly coating the sperm, and is also found in blood and mucus. Protein expression is reduced in PrCa, so its gradual loss is associated with the development of PrCa. Its expression is regulated by the polycomb group protein EZH2, and the expression of *MSMB* can be silenced by trimethylation. PSP94 has roles in growth regulation and the induction of apoptosis in PrCa cells and as it leaks into the blood, its serum level can be measured. As there is a tight correlation between the level of PSP94 and PrCa progression (Reeves *et al*, 2006; Bjartell, 2007), PSP94 is believed to be an independent predictor of recurrence of cancer after radical prostatectomy. Based on these observations it is clear that the regulation of the expression of *MSMB* is a key element in PrCa development and any sequence variant that has an effect on the level of gene expression is a good candidate for a causal variant.

In summary, we have not found any deleterious mutations in the coding sequences of *MSMB* in familial PrCa. This study has a 90% power to detect a rare mutation with a frequency of >1%. Based on this and other recent studies it is likely that SNP9, rs10993994, is the causative variant in the association of *MSMB* with PrCa risk. However, through resequencing, we have identified several new SNPs in the promoter region, which are also predicted to have some effect on splicing/transcription of the *MSMB* gene. A very rare new variant, whose effect cannot be statistically validated and rs41274660, a previously described variant are predicted to have a direct effect on prostate-specific TF-binding sites, one of which include androgen/progesterone/aldosterone receptors. Further functional studies are needed to fully establish the significance of these sequence variants individually or in combination in PrCa predisposition.

## Figures and Tables

**Table 1:** List of SNPs identified by re-sequencing of 192 familial prostate cancer cases

**SNP**	**NCBI 36 coordinates**	**dbSNP ID**	**Designation (VEGA transcipt OTTHUMG00000018212)**	**Genotype**	**No (of 192)**
SNP 1	10: 51218441	New	−1063 T>C	CT	1
SNP 2	10: 51218461	rs61847070	−1043 T>C	TC	34
				CC	1
SNP3	10: 51218615	New	−889 G>C	GC	4
SNP4	10: 51219036	New	−468 T>C	TC	1
SNP5	10: 51219205	rs12247790	−299 T>G	GG	1
SNP6	51219227^51219228	rs10669586	−276 indelCT		6
SNP7	10: 51219266	New	−238 C>T	TC	1
SNP8	10: 51219320	rs12770171	−184 C>T	CT	72
				TT	8
SNP9	10: 51219502	rs10993994	−2 T>C	TC	91
				TT	69
SNP10	10: 51219539	rs41274660	UTR −19 T>G	TG	7
				GG	1
SNP11	10: 51225699	New	IVS1 −38 T>G	TG	1
SNP12	10: 51225716	New	IVS1 −21 T>C	TC	1
SNP13	10: 51226117	rs2075894	IVS2 +275 T>C	TC	51
				CC	2
SNP14	10: 51226665	New	IVS2 −92 G>T	GT	2
SNP15	10: 51226682	New	IVS2 −75 G>T	GT	1
SNP16	10: 51226927	rs10994385	IVS3 +65 G>C	GC	52
				CC	1
SNP17	10: 51232109	New	IVS3 −168 C>T	CT	1

**Table 2:** (a) Common SNPs with significant difference in the frequency of alleles in 192 familial cases and 192 controls and (b) Haplotype analysis of the three common SNPs in the promoter region in 192 familial cases and 192 controls

**(a)**						
**SNP**	**NCBI 36 coordinates**	**SNP ID**	**Associated allele**	**Frequency in cases**	**Frequency in controls**	***P*-value**
**SNP2**	10: 51218461	ENSSNP10237085	C	0.094	0.048	0.0172
						
**SNP8**	10: 51219320	rs12770171	T	0.236	0.151	0.0036
						
**SNP9**	10: 51219502	rs10993994	T	0.453	0.352	0.0052
						
**(b)**						
**Haplotype**	**Frequency**	**Case, control ratio counts**	**Case, control frequencies**	***χ* ^2^**	***P*-value**	
SNP 2,8 and 9						
**TCC**	0.597	210.5 : 173.5, 234.6 : 127.4	0.548, 0.648	7.721	0.0055	
**TCT**	0.209	83.2 : 300.8, 72.9 : 289.1	0.217, 0.201	0.259	0.6111	
**TTT**	0.122	54.4 : 329.6, 36.4 : 325.6	0.142, 0.101	2.934	0.0868	
**CTT**	0.073	36.0 : 348.0, 18.1 : 343.9	0.094, 0.050	5.287	0.0215	

**Table 3:** Haplotype analysis of SNP 8 rs1277017 and SNP 9 rs10,993,994 using our data from stage1 and 2 genome-wide association study (GWAS) adjusted for strata (Eeles *et al*, 2008)

	**Haplotype**				
**N^a^**	**rs10993994**	**rs1277017**	***P*-value**	**Freq^b^**	**Odds ratio^c^ (OR)**
1	1 C	1 C		0.580	1
2	1 C	2 T	0.35	0.0021	1.38 (0.71–2.04)
3	2 T	1 C	7.0 × 10^−18^	0.210	1.35 (1.28–1.42)
4	2 T	2 T	3.7 × 10^−19^	0.208	1.37 (1.30–1.44)

a Haplotype number.

b Haplotype frequencies.

c Odds ratio and 95% confidence interval.
