# Supplementary material for: Mutation analysis of the MSMB gene in familial prostate cancer
Source: Br J Cancer. 2009 Dec 8;102(2):414–8. doi: 10.1038/sj.bjc.6605485 (PMC2816656; doi:10.1038/sj.bjc.6605485)
Supplement: Supplementary Table 1 [file 6605485x1.doc]

Suppl Table 1: Conservation around 17 SNPs showing (top) % match between 81mers aligned with DNA from different species, and showing conservation of the 3bp across the SNP itself (below)

|  | **Start** | **Stop** | **Conservation** | 81 bp % homol | | | | | | **Avg** | **Sum** | Notes |
| --- | --- | --- | --- | --- | --- | --- | --- | --- | --- | --- | --- | --- |
| **SNP#** |  |  | ***Species*** | **Chimp** | **Rhesus** | **Cow** | **Dog** | **Rat** | **Mouse** |  |  |  |
| 1 | 51218401 | 51218481 |  | 99 | 94 | 63 | 62 | - | - | **80** | 318 | LTR repeat |
| 2 | 51218421 | 51218501 |  | 100 | 95 | 62 | 72 | - | - | **82** | 329 | LTR repeat |
| 3 | 51218575 | 51218655 | *- = no data* | 100 | 93 | 51 | 62 | - | - | 77 | 306 | LTR repeat |
| 4 | 51218996 | 51219076 |  | 100 | 96 | 51 | 56 | 51 | 47 | 67 | 401 |  |
| 5 | 51219165 | 51219245 |  | 98 | 96 | 66 | 58 | 53 | 57 | 71 | 428 | rs12247790 |
| 6 | 51219187 | 51219267 |  | 98 | 93 | 64 | 41 | 55 | 54 | 68 | 405 |  |
| 7 | 51219226 | 51219306 |  | 98 | 90 | 73 | 46 | 58 | 58 | 71 | 423 | Prostate TF difference |
| **8** | 51219280 | 51219360 |  | 100 | 96 | 77 | 70 | 53 | 53 | 75 | **449** |  |
| 9 | 51219462 | 51219542 |  | 100 | 94 | 67 | 64 | 54 | 58 | 73 | 437 |  |
| 10 | 51219499 | 51219579 |  | 98 | 98 | 63 | 63 | 63 | 49 | 72 | 434 | Prostate TF difference |
| **11** | 51225659 | 51225739 |  | 99 | 98 | 72 | 67 | 65 | 67 | 78 | **468** | upstr MSMB |
| **12** | 51225676 | 51225756 |  | 99 | 95 | 88 | 69 | 69 | 69 | **82** | **489** | upstr MSMB |
| 13 | 51226077 | 51226157 |  | 96 | 94 | - | - | - | - | 95 | 190 | SINE repeat |
| 14 | 51226625 | 51226705 | *incomplete | 100 | 98 | 56 | -71* | - | - | 85 | 254 | at end of LINE repeat |
| 15 | 51226642 | 51226722 | -71* | 100 | 100 | 52 | -71* | - | - | 84 | 252 | ditto |
| 16 | 51226887 | 51226967 |  | 99 | 96 | 63 | 60 | 46 | - | 73 | 364 |  |
| 17 | 51232069 | 51232149 |  | 99 | 95 | 48 | 46 | 59 | 59 | 68 | 406 |  |
|  |  |  | Conservation of 3bp around SNP | * 1=common, 2=rare,( )=not conserved, 0=wrong base, -1=gap, +1=insertion, - =no alignment | | | | | |  |  |  |
| 1 |  |  | 3bp around | 1 | 1 | (1) | 1 | - | - |  |  | A common |
| 2 |  |  | SNP* | 1 | 1 | (1) | (1) | - | - |  |  | T common |
| 3 |  |  |  | 1 | 1 | (1) | (-1) | - | - |  |  | G common |
| 4 |  | * 1=common allele | | 1 | 1 | (0) | (1) | (-1) | (0) |  |  | T common |
| 5 |  | 2=rare allele | | 1 | 1 | (1) | 1 | 1 | 1 |  |  | T common |
| 6 |  | ()=not conserved, | | 2 | 2 | -1 | -1 | (0) | (0) |  |  | -/CT (- common) |
| 7 |  | 0=wrong base, | | 1 | 1 | (1) | 2 | (0) | (0) |  |  | C common |
| **8** |  | -1=gap, |  | 1 | 1 | (-1) | 1 | (-1) | (2) |  |  | C common |
| 9 |  | +1=insertion, | | 1 | 1 | 1 | (1) | (1) | (0) |  |  | T common |
| 10 |  | -=nothing aligned here | | 1 | 1 | (0) | 1 | (-1) | (+1) |  |  | T common |
| **11** |  |  |  | 1 | 1 | (1) | (1) | (1) | (1) |  |  | T common |
| **12** |  |  |  | 1 | 1 | 1 | 0 | 1 | (1) |  |  | T common |
| 13 |  |  |  | 2 | 2 | - | - | - | - |  |  | T common |
| 14 |  |  |  | 1 | 1 | (1) | 0 | - | - |  |  | G common |
| 15 |  |  |  | 1 | 1 | (2) | 1 | - | - |  |  | G common |
| 16 |  |  |  | 2 | 2 | 2 | 2 | -1 | - |  |  | G common |
| 17 |  |  |  | 1 | 1 | (2) | (2) | (1) | (1) |  |  | C common |
